# Current status and recent advances in resection cavity irradiation of brain metastases

**DOI:** 10.1186/s13014-021-01802-9

**Published:** 2021-04-15

**Authors:** Giuseppe Minniti, Maximilian Niyazi, Nicolaus Andratschke, Matthias Guckenberger, Joshua D. Palmer, Helen A. Shih, Simon S. Lo, Scott Soltys, Ivana Russo, Paul D. Brown, Claus Belka

**Affiliations:** 1 Department of Medicine, Surgery and Neurosciences, University of Siena, Policlinico Le Scotte, 53100 Siena, Italy; 2 IRCCS Neuromed, Pozzilli, IS Italy; 3 Department of Radiation Oncology, University Hospital, LMU Munich, Munich, Germany; 4 German Cancer Consortium (DKTK), Partner Site Munich, Munich, Germany; 5 Department of Radiation Oncology, University Hospital of Zurich, University of Zurich, Raemistrasse 100, 8091 Zurich, Switzerland; 6 Department of Radiation Oncology, Arthur G. James Cancer Hospital, The Ohio State University, Columbus, OH USA; 7 Department of Radiation Oncology, Massachusetts General Hospital, Boston, MA USA; 8 Department of Radiation Oncology, University of Washington School of Medicine, Seattle, WA USA; 9 Department of Radiation Oncology, Stanford University, Stanford, CA USA; 10 Radiation Oncology Unit, University of Pittsburgh Medical Center Hillman Cancer Center, San Pietro Hospital FBF, Rome, and Villa Maria Hospital, Mirabella, AV Italy; 11 Department of Radiation Oncology, Mayo Clinic, Rochester, MN USA

**Keywords:** Stereotactic radiosurgery, Hypofractionated stereotactic radiotherapy, Resection cavity, Brain metastases, Radiation necrosis

## Abstract

Despite complete surgical resection brain metastases are at significant risk of local recurrence without additional radiation therapy. Traditionally, the addition of postoperative whole brain radiotherapy (WBRT) has been considered the standard of care on the basis of randomized studies demonstrating its efficacy in reducing the risk of recurrence in the surgical bed as well as the incidence of new distant metastases. More recently, postoperative stereotactic radiosurgery (SRS) to the surgical bed has emerged as an effective and safe treatment option for resected brain metastases. Published randomized trials have demonstrated that postoperative SRS to the resection cavity provides superior local control compared to surgery alone, and significantly decreases the risk of neurocognitive decline compared to WBRT, without detrimental effects on survival. While studies support the use of postoperative SRS to the resection cavity as the standard of care after surgery, there are several issues that need to be investigated further with the aim of improving local control and reducing the risk of leptomeningeal disease and radiation necrosis, including the optimal dose prescription/fractionation, the timing of postoperative SRS treatment, and surgical cavity target delineation. We provide a clinical overview on current status and recent advances in resection cavity irradiation of brain metastases, focusing on relevant strategies that can improve local control and minimize the risk of radiation-induced toxicity.

## Introduction

Brain metastases are a common and devastating complication of cancer. Surgical resection remains an effective treatment for brain metastases, especially for larger lesions causing mass effect and consequentially serious neurological symptoms. Postoperative whole brain radiation therapy (WBRT) has been traditionally employed in patients with resected brain metastases owing to its efficacy in reducing the risk of local recurrence in the surgical bed and the incidence of new distant metastases [1]. Stereotactic radiosurgery (SRS), which is the recommended treatment for patients with a limited number of brain metastases [2, 3], has been increasingly employed to target the postoperative resection cavity as an alternative to WBRT [4–6]. Several retrospective series of stereotactic irradiation given as single fraction, referred to as SRS, or delivered in few fractions, typically named hypofractionated stereotactic radiotherapy (HSRT) or fractionated SRS, have shown local control rates from 70 to 90% at one year with low incidence of radiation-induced toxicity [4, 5]. Data from two randomized trials [7, 8] have demonstrated that (1) SRS to the resection cavity significantly reduces bed recurrence rates compared with observation alone [8], and (2) decreases the risk of cognitive decline in patients with brain metastases as compared to WBRT, without diminishing survival [7].

Based on this accumulated evidence, this approach has become the recommended treatment following surgical resection of a brain metastasis. However, optimal management of resected brain metastases remains challenging and several issues remain to be resolved, including the timing of postoperative SRS treatment, optimal radiation dose prescription and fractionation, and target delineation of the surgical bed [9].

We provide a critical overview on current status and recent advances in resection cavity irradiation of brain metastases, with the aim of answering questions relevant to clinical and technical issues, such as the appropriate radiation technique, optimal radiation schedule, risk of leptomeningeal disease and treatment-related toxicity for patients receiving radiotherapy to postoperative resection cavity of brain metastases.

## Methods and materials

A literature search was conducted in MEDLINE PubMed using combinations of the following medical subjects headings (MeSH) and free-text words: “radiotherapy” or “radiosurgery” and “resection”, “brain metastasis”, “postoperative”. We included clinical trials, retrospective studies, and review articles that were published within the past 15 years to reflect modern systemic therapies and neurosurgical and radiosurgical techniques. Articles were selected if they had (1) 1-year local control and/or rates of radiation-induced brain necrosis reported and (2) radiosurgery administered as definitive or postoperative treatment. Articles were excluded from the review if they had a non-English abstract, were not available through Pubmed, were pediatric series or case studies involving less than 30 patients, or were duplicated publications. To identify additional articles, the references of articles identified through the formal searches were scanned for additional sources. Based on the initial searches, a total of 352 articles were identified. Finally, 69 papers containing relevant data on clinical outcomes following postoperative SRS/HSRT in adult patients were chosen for this review.

## Results

### Local control and survival

The clinical success of SRS in patients presenting with a limited number of brain metastases resulted in its application to surgical cavities as an alternative to WBRT. Several retrospective studies reported local control and overall survival rates of 70% to 90% and of 50% to 70% at 12 months, respectively, following either SRS (Table 1) [7, 8, 10–23] and HSRT (Table 2) [24–37]. The question on the efficacy and safety of postoperative SRS has been recently addressed in two randomized trials comparing postoperative SRS to observation or to WBRT, respectively [7, 8].

**Table 1:** Selected studies of postoperative stereotactic radiosurgery (SRS) to surgical bed

References	Pts (No)	Date	Interval between surgery/SRS	SRS modality	Median SRS dose (Gy)	PTV (cc)	GTV-to-PTV margins	Median follow-up (months)
Jensen et al. [10]	106	2001–2009	3.5 w	GK	17 (11–23)	12.6 (1.2–74.0)	1 mm	NR
Rwigema et al. [11]	77	2005–2010	3–4 w	CK	12–27 in 1–3 fr (SRS 70%)	7.6 (0.5–59)	1 mm	13.8
Prabhu et al. [12]	62	2007–2010	4.5 w	LINAC	18	13.9 (1.6–80)	0–2 mm; > 1 mm (95%)	12.4
Robbins et al. [13]	85	2000–2011	within 8 w	LINAC	16	13.95	2–3 mm	11.2
Luther et al. [14]	120	2002–2012	4 w	GK	16	8	2–3 mm	12.6
Brennan et al. [15]	49	2208–2009	4 w (2–8)	LINAC	15–22	≥ 3 cm, 18 < 3 cm, 32	2 mm	12
Iorio-Morin et al. [16]	110	2004–2013	> 3w (60pts), < 3w (53pts)	GK	18	12 (0.6–43)	1 mm	10
Ojerholm et al. [17]	91	2007–2013	6 w	GK	16	9.2	0 mm	9.8
Abel et al. [18]	85	2003–2013	3–6 w	GK	17.3 (14–20)	12 (0.3–83)	0 mm	16.4
Eaton et al. [19]	75	2007–2014	NR	LINAC	SRS 15 (39 pts) HSRT 24–30/3–5 fr (36 pts)	SRS 20.5 HSRT 37.7	1.5–2 mm	15
Strauss et al. [20]	100	2005–2013	4 w	LINAC	20	4 ± 3.1 (mean)	0 mm	16.3 (mean)
Johnson et al. [21]	112	2006–2013	2–4 w	GK	16	9.85 (0.9–41.1)	0 mm	9
Rava et al. [22]	87	2002–2010	4 w	GK	18	13.4 (3–40.8)	1–2 mm	7.1
Brown et al. [7]	194	2011–2015	Within 4 w	LINAC/ GK/CK	SRS (12–18); WBRT (37.5/15)	NR	2 mm	11.1
Mahajan et al. [24]	128	2009–2016	Within 4 w	GK	16 (12–18)	NR	1 mm	11.1
Bachmann et al. [23]	75	2010–2015	NR	LINAC	SRS 18 HSRT 40/10fr	SRS 8.4; HSRT 22.6	1 mm	11.2

References	1-Year LC (%)	1-Year DP (%)	1-Year OS (%)	BED Gy_10_ (Tumor)	BED Gy_2_ (normal brain)	LMD (%)	Symptomatic RN (%)
Jensen et al. [10]	80.3	64.6	46.8	45.9 (23.1–75.9)	161.5 (71.5–287.5)	7	2.7
Rwigema et al. [11]	76	46	62.5	SRS 37.5 HSRT 51.3	SRS 127.5 HSRT 148.5	1.3	3
Prabhu et al. [12]	78	51	70	50.4	180	NR	8 at 1 year
Robbins et al. [13]	81.4	58.1	51.5	41.6	144	NR	8
Luther et al. [14]	87	40	63	41.6	144	NR	7.5
Brennan et al. [15]	78	44	50	37.5–70.4	127.5–264	NR	17.5
Iorio-Morin et al. [16]	< 3w 87 > 3w 61	54	63	50.4	180	11	22
Ojerholm et al. [17]	81	27	45	41.6	144	NR	7
Abel et al. [18]	87	52	65	47.2 (33.6–60)	166.9 (112–220)	NR	8
Eaton et al. [19]	SRS 27.2 HSRT 25.6	52	SRS 41.1 HSRT 54.9	SRS 37.5 HSRT 43.2–48	SRS 127.5 HSRT 120–126	NR	SRS 6.8 HSRT 16.4
Strauss et al. [20]	84	42	63.5	60	220	9.8	5
Johnson et al. [21]	84.4	50	12.9	41.6	144	16.9 at 1 year	NR
Rava et al. [22]	82	44	54	50.4	180	NR	10.3
Brown et al. [7]	SRS 60.5 WBRT 80·6 (*p* = 0.00068)	SRS 35 WBRT 11 (*p* = 0.0004)	SRS 50 WBRT 48	SRS 26.4–50.4 WBRT 46.8	SRS 84–180 WBRT 84.4	SRS 7.2 WBRT 6.4	4 (all after SRS)
Mahajan et al. [24]	SRS 57 OBS 28 (*p* = 0.0015)	SRS 58 OBS 67	SRS 65 OBS 63	41.6 26.4–50.4	144 84–180	SRS 28 OBS 16	NR
Bachmann et al. [23]	72	40	64	SRS 50.4 HSRT 56	SRS 180 HSRT 120	NR	22

LINAC, linear accelerator; GK, Gamma Knife; CK, CyberKnife; SRS, stereotactic radiosurgery; WBRT, whole brain radiation therapy; HSRT, hypofractionated stereotactic radiation therapy; OBS, observation; p, prospective; BED Gy10, biological equivalent dose with an α/β ratio of 10 Gy; BED Gy2, biological equivalent dose with an α/β ratio of 2 Gy; w, weeks; LC, local control; DP, dostant progression; OS, overall survival; NR, not reported

**Table 2:** Selected studies of postoperative hypofractionated stereotactic radiotherapy (HSRT) to surgical bed

References	Pts (No)	Date	Interval between surgery/SRS	SRS modality	Median dose (Gy)/fractions	PTV (cc)	CTV/PTV margins (mm)	Median follow-up (months)
Minniti et al. [24]	101	2005–2012	3 w	LINAC	27/3 fr	29.5 (18.5–52.7)	2 mm	16
Ahmed et al. [25]	65	2009–2013	5 w (1–15)	LINAC	25–30/5 fr	16.88 (4.9–128.4)	1–2 mm	8.5
Keller et al. [26]	187	2008–2015	6.5 w	LINAC	33/3 fr	14.15 (0.8–65.8)	2 mm	15
Minniti et al., [27]	60	2008–2015	3 w	LINAC	27/3 fr	20.6 (6.1–66.8)	CTV, 1 mm; PTV, 1 mm	13
Zhong et al. [28]	117	2006–2015	2–4 w	LINAC	15–30 Gy/1–5 fr	SRS 18.6 HSRT 33.7	0–3 mm	22
Jhaveri et al. [29]	133	2006–2016	3–4 w	LINAC	30–35/5 fr	14.7 (1 mm) 20.3(> 1 mm)	1 mm (25.2%) > 1 mm (74.8%)	17.7
Minniti et al. [30]	95	2011–2017	3 w	LINAC	27/3 fr	22.4 (6.3–67.4)	CTV, 1 mm PTV, 1 mm	13
Navarria et al. [31]	101	2015–2018	3–4 w	LINAC	30/3 fr	52.9 (7.6–282.9)	2 mm	26
Soliman et al. [32]	122	2009–2014	3–4 w	LINAC	30/5 fr	30.1	2 mm	16
El Shafie et al. [33]	101	2015–2019	5.1 w	CK SRS/ HSRT (50); WBRT (51)	30–35/5 fr	18.2	2 mm	22.8
Faruqi et al. [34]	118	NR	6 w	LINAC	25–35/5 fr	24.9	2 mm	12
Garimall et al. [35]	134	2012–2018	4 w	LINAC	24/3 fr (most frequent)	28 (2.4–149.2)	1–2 mm	14
Eitz et al. [36]	558	2003–2019	5 w (4–6)	LINAC	30/5 fr (most frequent)	23.9 (13.5–36.3)	2–3 mm	12.3
Shi et al. [37]	422	2007–2018	3 w (2–4)	CK	24–27/3 fr (85%); SRS 16–18 (15%)	14.3 (9.1–22.2)	1–3 mm (76%) 0 mm (24%)	10.1

References	1-Year LC (%)	1-Year DP (%)	1-Year OS (%)	BED Gy_10_ (Tumor)	BED Gy_2_ (normal brain)	LMD (%)	Symptomatic RN (%)
Minniti et al. [24]	93	50	69	51.3	148.5	8	9 (7 and 16 at 1 and 2 years)
Ahmed et al. [25]	87	49.1	65.2	37.5–48	87.5–120	10	1.5
Keller et al. [26]	88.2	39.3	62.5	69.3	214.5	10.6% at 1 year	15.4 at 1 year
Minniti et al., [27]	88	45	58	51.3	148.5	9	15 (13 at 1 year)
Zhong et al. [28]	87.7 (size ≤ 4 cm) 84 (size > 4 cm)	46.7 (size ≤ 4 cm) 55 (size > 4 cm)	80.6 (size ≤ 4 cm) 67.6 (size > 4 cm)	SRS 46.8 HSRT 35.7–49.2	SRS 161.5 HSRT 87.5–147.5	13.1 (≤ 4 cm) 15.1 (> 4 cm)	26.9 (≤ 4 cm) 28.4 (> 4 cm)
Jhaveri et al. [29]	85	45	NR ( median 15.6 months)	48–59.5	120–157.5	12.8	21.1 (16.7 at 1 year)
Minniti et al. [30]	88	50	59	51.3	148.5	7	12 at 1 year
Navarria et al. [31]	85.9	32	81.9	60	180	8.9	25.7 (5.9 grade 3)
Soliman et al. [32]	84	45	62	48	120	22	6
El Shafie et al. [33]	SRS/HSRT 94.9 WBRT 81.7	SRS/HSRT 42 WBRT 35	SRS/HSRT 80 WBRT 50 (*p* < 0.002)	48–59.5	120–157.5	8	6
Faruqi et al. [34]	84	NR	62	37.5–49.5	87.5–157.5	NR	7.6
Garimall et al. [35]	92	NR	NR	43.2	120	NR	2
Eitz et al. [36]	84	45	65	48	120	13.1	8.6
Shi et al. [37]	93.5	44	13.9	HSRT 43.2–51.3; SRS 41.6–50.4	HSRT 120–148.5; SRS 144–180	15.8 (13 at 1 year)	9

LINAC, linear accelerator; GK, Gamma Knife; CK, CyberKnife; SRS, stereotactic radiosurgery; WBRT, whole brain radiation therapy; HSRT, hypofractionated stereotactic radiation therapy; OBS, observation; BED Gy10, biological equivalent dose with an α/β ratio of 10 Gy; BED Gy2, biological equivalent dose with an α/β ratio of 2 Gy; w, weeks; LC, local control; DP, dostant progression; OS, overall survival; NR, not reported

Mahajan et al. [8] compared adjuvant SRS to observation in 128 patients who underwent gross total resection for 1–3 brain metastases between 2009 and 2016 at The University of Texas M.D. Anderson Cancer Center. The primary endpoint was the local tumor-free recurrence rate. The target volume was defined as the surgical cavity on the volumetric MR imaging with an additional margin of 1 mm. Prescription doses were 16, 14, and 12 Gy for target volumes of ≤ 10 cc, 10.1–15 cc, and > 15 cc, respectively, given in a single session by Gamma Knife. The 12-month tumor-free recurrence rates were 43% in the observation group and 72% in SRS group (*p* = 0.015), with comparable median overall survival times of 18 and 17 months. Amongst cavities treated with SRS, metastasis size was a significant predictor of local failure; 12-month local control rates were 91% for patients with tumors with a maximal diameter of ≤ 2.5 cm, 40% for patients with tumors > 2.5 to 3.5 cm in diameter, and 46% for patients with tumors > 3.5 cm in diameter. Considering that larger tumors received radiation doses of ≤ 14 Gy, these data indicate that lower SRS doses applied in patients with larger resection cavities, corresponding to a biological effective dose assuming an α/β ratio of 10 Gy for the tumor (BED_10Gy_) < 33.6 Gy (Table 3), may be not sufficient to control microscopic disease. In addition, the trial confirmed previous evidence that surgical resection alone is insufficient to provide satisfactory local control [1, 2] despite improvements in neurosurgical techniques, such as stereotactic navigation and cortical mapping.

**Table 3:** Biological equivalent dose (BED) and equivalent dose in 2 Gy per fraction (EQD2) for various SRS/HSRT radiation schedules

Dose regimen	BED Gy_10_ (Tumor)	EQD10/2	BED Gy_2_ (Brain Parenchyma)	EQD2/2
30 Gy/5 fractions	48	40	120	60
25 Gy/5 fractions	37.5	31.25	87.5	43.75
27 Gy/3 fractions	51.3	42.75	148.5	74.25
24 Gy/3 fractions	43.2	36	120	60
20 Gy/1 fraction	60	50	220	110
18 Gy/1 fraction	50.4	42	180	90
16 Gy/1 fraction	41.6	34.67	144	72
14 Gy/1 fraction	33.6	28	112	56
12 Gy/ 1 fraction	26.4	22	84	42

BED Gy10, biological equivalent dose with an α/β ratio of 10 Gy; BED Gy2, biological equivalent dose with an α/β ratio of 2 Gy; EQD10/2, eqivalent dose in 2 Gy/fractions with a BED Gy10; EQD2/2, eqivalent dose in 2 Gy/fractions with a BED Gy2; SRS, stereotactic radiosurgery; HSRT, hypofractionated stereoactic radiation therapy

In the NCCTG N107C/CEC.3 prospective randomized trial of 194 patients with one resected brain metastasis and a resection cavity less than 5 cm in maximal size who were randomly assigned to either SRS (12 to 20 Gy) to WBRT (30–37.5 Gy in 10–15 daily fractions), Brown et al. [7] reported superior preservation of neurocognitive function and quality of life in patients who received SRS with no negative impact on survival, although adjuvant WBRT was associated with better intracranial control compared to SRS. With similar median survival times of 12.2 months in the SRS arm and 11.6 months in the WBRT arm, median cognitive deterioration-free-survival was longer in patients randomized to SRS at both 3 and 6 months, reaching statistical significance for immediate memory (*p* = 0.00062), delayed memory (*p* = 0.00054), processing speed (*p* = 0.023), and executive function (*p* = 0.015). The negative impact of WBRT on cognitive function, quality of life and functional independence remained persistent over time. The prescribed SRS dose was selected based on surgical cavity volume: 20 Gy if the cavity volume was less than 4.2 ml, 18 Gy if 4.2–7.9 ml, 17 Gy if 8–14.3 ml, 15 Gy if 14.4–19.9 ml, 14 Gy if 20–29.9 ml, and 12 Gy if 30 ml or more up to the maximal surgical cavity extent size of 5 cm. An unexpected finding from this trial was an inferior surgical bed control rate for patients treated with postoperative SRS as compared to those who received WBRT; the 6- and 12-month estimates of surgical bed control were 80.4% and 60.5% with SRS versus 87.1%, and 80.6% with WBRT (*p* = 0.00068). Nevertheless, the study confirms results observed in other phase III trials of intact brain metastases [2, 3] and suggests that adjuvant SRS should be considered the recommended treatment for surgical bed because of significantly lower risk of cognitive decline and better quality of life compared to WBRT [3, 38].

While these randomized studies reported on single-fraction SRS, similar results have been observed following HSRT using different dose and fractionation schedules, typically 24–27 Gy given in three fractions or 25–30 Gy given in 5 fractions (Table 2). Surveillance imaging following both SRS and HSRT to the resection cavity is important for the increased risk of distant brain failure after focal irradiation as opposed to WBRT. Therefore, frequent magnetic resonance imaging (MRI), typically at regular intervals of 2–3 months after SRS, is strongly recommended.

Several studies have evaluated the impact of different prognostic factors on local tumor control following radiation to the resection cavity. Larger preoperative tumor size and cavity volumes greater than 3 cm [7, 8, 12, 14, 15, 26, 29, 39–41], incomplete resection [18, 33, 41, 42], lower radiation dose [14, 16, 23, 40, 41, 43], pretreatment tumor volume in contact with dura [15, 26, 44], and longer interval time between surgery and radiation treatment [16, 45, 46] have been significantly correlated with worse local control. Factors associated with longer survival include Karnofsky Performance Status (KPS) score of 80% or greater, an interval less than 4 weeks between resection and postoperative radiation treatment [45, 46], and a controlled primary tumor [8, 24, 30, 36]. In contrast, combined systemic treatment and histology did not emerge as independent prognostic factors for either local control or survival in most studies [8, 23, 25, 35, 36, 41]**.**

### Optimal dose and fractionation

Tables 1 and 2 show patient data and clinical outcomes of postoperative radiation to the resection cavity given as SRS or HSRT. Currently, there are several terms that have been used interchangeably for fractionated SRS, including multi-fraction, multi-dose, multi-session SRS, and hypofractionated stereotactic radiotherapy (HSRT) where dose is generally delivered in few, generally 2–5, fractions using frameless, mask-based SRS systems with the same level of accuracy of fixed-frame SRS [47]. Using single-fraction SRS with doses of about 12 to 20 Gy, 16 studies including 1,556 patients show median local control and overall survival rates of about 60–90% and 50–70% at 12-months, respectively (Table 1). For 1.749 patients receiving HSRT as postoperative treatment included in 13 studies (Table 2), 12-month local control and overall survival rates were 88–95% and 58–82% using 24–33 Gy in 3 fractions, respectively, and 84–95% and 62–77% using 25–35 Gy in 5 daily fractions, respectively. Median cavity volumes were 12.7 ml (0.9–83 ml) for patients receiving postoperative SRS and 23.8 ml (2.8–283 ml) for those treated with HSRT. In a recent analysis of 588 resection cavities treated with postoperative irradiation included in nine studies, Lehrer et al. found no significant differences in the estimated 12-month local control between single-fraction SRS and fractionated SRS (68% vs 86.8%; *p* = 0.1); however, larger cavities were more likely to receive fractionated treatment.

A significant correlation between the radiation dose and local control has been observed for both SRS and HSRT [7, 8, 14, 16, 23, 35, 40, 42, 43]. In the Mahajan trial [8], SRS prescription doses were 16, 14, and 12 Gy for target volumes of ≤ 10 ml, 10.1–15 ml, and > 15 ml, respectively, given in a single session by Gamma Knife. Local control rates at 12 months of resection cavities were 91% for 40 patients with tumors with a maximal diameter of ≤ 2.5 cm receiving 16 Gy and 46% for 33 patients with tumors > 3.5 cm in diameter receiving 12 Gy (*p* = 0.0002). In the Brown trial [7], local control decreased for postoperative cavity volumes > 20 ml who received radiation doses < 15 Gy, being significantly lower than that observed after WBRT. A significantly better local control with radiation dose ≥ 18 Gy has been observed in other retrospective studies [14, 16, 23, 42]. It needs to be added that data discussed above do not allow a separate analysis of SRS dose versus volume, as larger volumes were consistently treated with lower SRS doses because of concerns of toxicity.

For HSRT, most common schedules were 24–27 Gy in 3 fractions and 30–35 Gy in 5 fractions with a reported similar 12-month local control of about 85–95%, as shown in Table 2; in contrast, lower doses, such as 5 × 5 Gy or 3 × 7 Gy, were associated with lower local control [35, 40, 43]. In a retrospective study of 39 patients with 43 surgical beds treated with postoperative HSRT, Kumar et al. [49] found that 30 Gy in 5 fractions and 27 Gy in 3 fractions provided better local control (93–100%) compared to lower dose 3- and 5-fraction regimens. Using the linear quadratic model to compare radiation doses of different fractionation schedules to predict tumor control probability and normal tissue complication probability [50], available data indicate that BED_10Gy_ > 40 Gy should be delivered to the surgical bed to achieve excellent local control. Calculation of BED_10Gy_ for the tumor and BED_2Gy_ for brain parenchyma with respective equivalent doses in 2 Gy fractions (EQD2_2_) using different dose and fractionation is shown in Table 3. In the respect of healthy tissue constraints, this means in clinical practice that radiation doses greater than 16 Gy given as single fraction, 24 Gy given in 3 fractions, and > 27.5 Gy given in 5 fractions should be recommended to improve local cavity control, especially in patients with radioresistant tumors.

### Cavity volume dynamics and timing of treatment

Target delineation of a resected brain metastasis is typically represented by the rim of enhancement at the edge of the resection cavity. While accurate contouring can be performed using thin-slice contrast-enhanced T1-weighted MRI sequences, the challenge is that the surgical bed is dynamic after surgery and prone to significant changes in resection cavity dimensions before SRS treatment, subsequently increasing the risk of missing the target and delivering unnecessary high radiation doses to surrounding normal brain parenchyma. Several, but not all, studies reporting on dynamic changes of resection cavity aiming to define the optimal SRS treatment timing indicate a postoperative decrease of the cavity volumes [25, 46, 51–54]. In a series of 57 patients who received postoperative SRS to the resection cavity, Scharl et al. [54] found significantly average cavity-volume reduction of 23.4% occurring between immediate post-resection MRI and planning MRI (*p* < 0.01). Regardless of the initial volume, cavity shrinkage occurred in 79.1%, remained stable in 3.5%, and increased in 17.4% of cases at a median time of 4 weeks after surgery. In another series of 59 patients with 61 cavities treated with postoperative SRS to the resection cavity, Alghamdi et al. [51] found an average cavity volume reduction of 22.5% at a median time of 4 weeks after surgery, with most changes occurring within 3 weeks. Tumor size > 3 cm, dural involvement and longer time from surgery were significant predictors of cavity volume reduction. Overall, an average cavity volume reduction of 15% to 43% has been reported in several published studies [46, 52, 53, 55], with larger tumor cavities (> 3 cm) that are associated with greater reduction. With regard to the timing, cavity volume reduction occurs within the first 3–4 weeks after surgery in 58–90% of resected brain metastases [25, 46, 51, 53, 54]; however, no change or increase in cavity size have been reported in few studies in the first 3–4 weeks [46, 52, 55].

The reported high incidence of significant changes in the postoperative resection cavity raises the question of the optimal timing for SRS treatment. As the majority of studies indicate that shrinkage occurs consistently over time in a significant proportion of patients, waiting a few weeks to perform SRS may represent an effective strategy to treat a smaller cavity volume, possibly limiting the risk of neurological toxicity while maintaining the efficacy of treatment. However, longer intervals more than 3–4 weeks between surgery and radiation treatment should be avoided because they have been associated with an increased risk of worse local control [16, 20, 39, 56]. In a retrospective series of 110 patients with 113 cavities treated with postoperative Gamma Knife SRS with a marginal dose of 18 Gy, Iorio-Morin et al. [16] reported local control rates of 73% at 12 months. Lower maximum radiation dose and a surgery-to-SRS delay longer than 3 weeks were risk factors for local recurrence. The estimated 12-month control rates dropped from 87 to 61% if SRS was performed more than 3 weeks after resection. This difference in rates of surgical bed control remained throughout follow-up; at 36 months, the group that received SRS less than three weeks after surgery had a 72% rate of local control compared to 46% for patients who received SRS more than three weeks after surgery. A possible explanation is that a longer delay might lead to an increased spread of microscopic disease that is harder to target because it is not yet radiographically evident. In this regard, other studies have observed a significant correlation between increasing delay between surgery and SRS and local failure [10, 20, 57, 58]. Overall, the median interval reported in the majority of studies of either SRS or HSRT was 19 days, with few exceptions of patients exceeding 5–6 weeks. Even though protocols are different with regard to technique, dose fractionation, and interval between surgery and radiation treatment, there is a general consensus to perform postoperative SRS/HSRT to the resection cavity within maximum four weeks after surgery with planning MRI acquired < 7 days before treatment to limit negative impact of cavity changes on clinical outcomes.

### Target volume delineation and margins

**Fig. 1:**
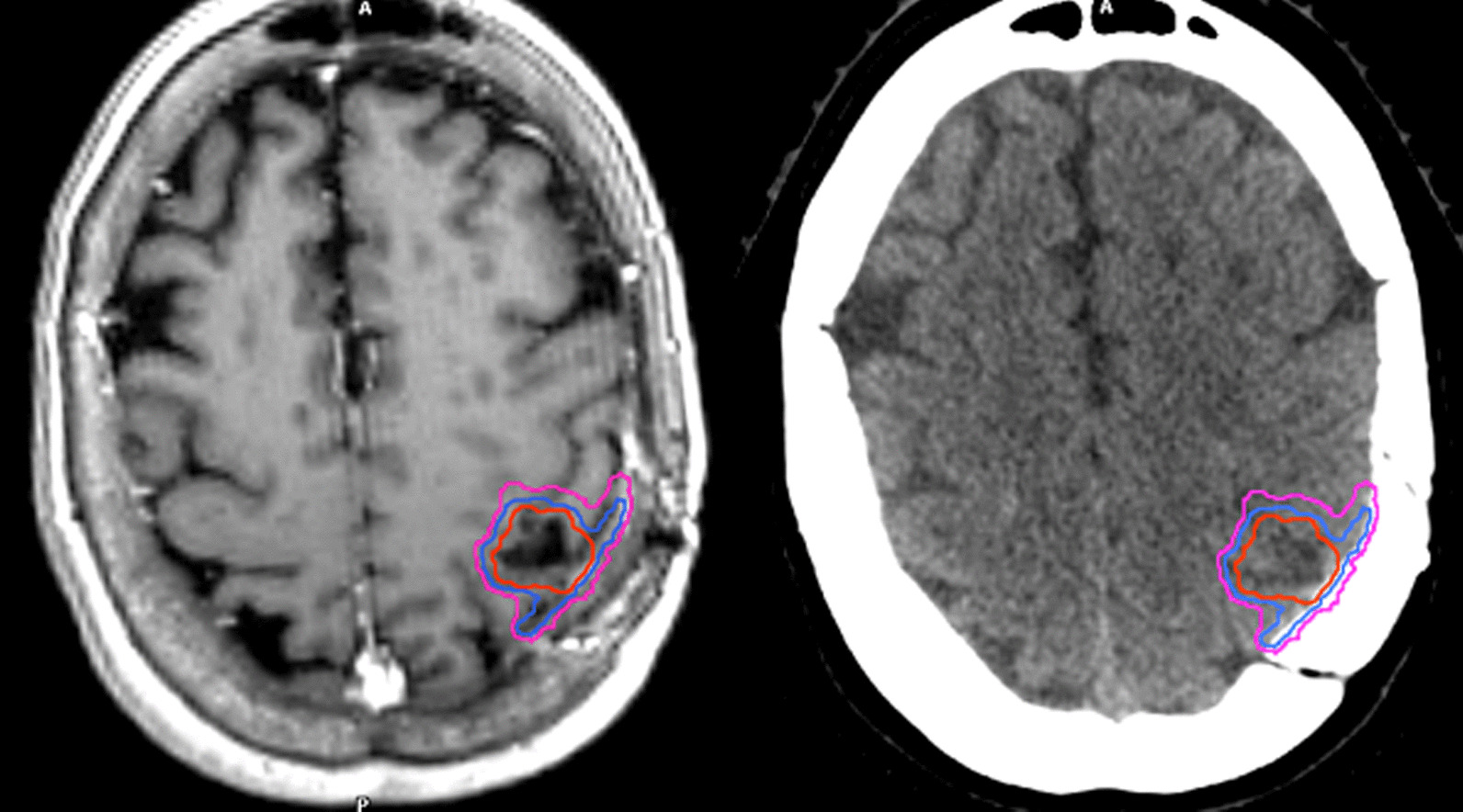
An overview of target volumes for postoperative resection cavity is presented on post-contrast T1-weighted MRI sequences and CT scans. The gross tumor volume (GTV) is presented in red, the clinical target volume (CTV) in blue and the planning tumor volume (PTV) in pink. For this case, CTV was created by 1-mm expansion of the GTV, extended by 5 mm along the bone flap beyond the initial region of preoperative tumor contact. Note that an extension by 10 mm along the meningeal margin for brain metastases with preoperative dural contact [59] or the inclusion of the entirety of the craniotomy site [71] has been suggested by some authors

Target delineation of the resection cavity remains challenging and has not yet been defined, especially in the setting of large volumes. This may be the reason that in some studies, long-term local control after SRS has been found to be worse compared to post-resection WBRT [7]. In a recently published consensus guideline on target delineation of the postoperative cavity, the primary recommendations for CTV delineation using contrast-enhancing T1-weighted MRI scan include contouring of the entire surgical cavity with the exclusion of vasogenic edema and include a margin up to 5 mm along the bone flap/meningeal margin [59]. For tumors in contact with the dura preoperatively, the guidelines recommend a GTV-to-CTV margin up to 10 mm along the bone flap beyond the initial region of preoperative tumor contact. An example of target delineation is shown in Fig. 1 (to be chosen). In a study from University of California San Francisco of 58 patients with 60 resection cavity who received postoperative SRS by Sukso et al. [44], preoperative dural contact increased recurrence rate after postoperative SRS and the median distance of marginal recurrences from the target volume was 3 mm, supporting the CTV delineation consensus guidelines. Of note, the addition of a 10-mm dural margin increased the target volume overlap with the recurrence contours for 10 of the 14 recurrences. Further recommendations for CTV delineation comprise the inclusion of the entire surgical tract and a margin of 1 to 5 mm along the sinus for those tumors that were in contact with a venous sinus preoperatively.

These consensus guidelines provide suggestions for standardized postoperative cavity contouring indicating that target definition should be guided by both the preoperative volume and location of lesion and also the postoperative changes as seen at MRI scan; however, controversies continue to exist. In this regard, the use of further margins beyond the contouring for the surgical cavity remains to be defined. Use of margins may improve target coverage and compensate contouring inaccuracy, but SRS to large treatment volumes can be associated with an increased risk of radiation necrosis [5, 24, 29, 48]. In most studies, margins of 0 to 3 mm for GTV/CTV expansion provide equivalent 1-year local tumor control rates with no evidence of a significantly increased risk of radiation necrosis after either SRS (Table 1) or HSRT (Table 2); in contrast, a few studies suggested that the use of margins of 2 mm is associated with better local control [14, 40, 60]. Another controversial issue is the inclusion of surgical access track. Several studies did not include the surgical tract for deep lesions [7, 15, 24, 27, 31, 32, 36, 37, 61, 62]. In a series of 64 patients with 66 cavities receiving postoperative SRS for a resected brain metastasis with or without inclusion of surgical corridor in the CTV, Shi et al. [37] showed that omitting the surgical corridor was not associated with statistically significant differences in corridor or cavity recurrence or adverse radiation effects. Overall, current recommendations for accurate target delineation of postoperative resection cavity include the use of thin-sliced contrast-enhancing T1-weighted MRI with the inclusion of generous meningeal margins up to 1 cm in the CTV without any area of the surrounding edema. To accurately identify the preoperative tumor extent and dural involvement, preoperative contrast-enhanced T1-weighted MRI is preferred. The impact of different target volume delineation and margins in terms of local control and increased risk of radiation necrosis remains to be defined.

### Risk of leptomeningeal disease

Leptomeningeal disease (LMD) is defined as the spread of tumor cells within the leptomeninges and the subarachnoid space and occurs approximately in up to 10% of patients with solid cancer during the course of disease, commonly in the context of progressive systemic disease [63]. The diagnosis of leptomeningeal metastases can be challenging. It is based on clinical evaluation, cerebrospinal MRI and cerebrospinal fluid (CSF) analysis [61]. The classification of leptomeningeal metastasis considers also the imaging presentation which guides clinical decision-making independently of the identification of tumor cells in the CSF. MRI abnormalities of LMD include enhancement of the leptomeninges of the brain or spinal cord identified as enhancement of the cranial nerves and spinal nerve roots, brain surface, cerebellar folia, and within cerebral sulci.

A risk of LMD development up to 28% has been observed after surgical resection and adjuvant postoperative SRS/HSRT of brain metastases. Recent series observed an incidence of 6–15% at one year (Tables 1, 2), although most studies did not include data on the risk of LMD. Its development is presumably related to iatrogenic dissemination of tumor cells into cerebrospinal fluid and meninges at the time of resection, resulting in nodule forming subsequently. The variable risk reported across all studies may depend on differences in tumor histology, tumor size and location, pial involvement, and type of surgical resection. In addition, differences in imaging follow-up and discordance in physicians’ assessment of LMD are potential factors that explain such variable incidence. For example, the reported risk of LMD observed in Mahajan [8] and Brown [7] randomized trials were 28% and 7%, respectively, suggesting diagnostic variability. Factors associated with the development of LMD include breast cancer histology [17, 21, 29, 52, 64], posterior fossa location [5, 17], multiple brain metastases [21], type of surgical resection (piecemeal instead of “*en bloc*”) [65, 66].

An important finding that emerged from some studies is a peculiar pattern of the meningeal spread after postoperative cavity radiation. In a retrospective series of 1,188 patients with newly diagnosed brain metastases managed with neurosurgical resection and stereotactic radiation (n = 318) or radiation alone (n = 870), Cagney et al. [67] examined two patterns of intracranial recurrence: (1) the “classical” LMD, defined as subarachnoid enhancement involving the sulci of the cerebral hemispheres, cranial nerves, brainstem, cerebellar folia, or ependyma, and (2) pachymeningeal seeding, defined as nodular, enhancing tumors stemming from the pachymeninges (dura and/or outer arachnoid) extending 1 cm beyond the planning target volume of the stereotactic field. They found that resection was associated with pachymeningeal seeding (36 of 318 patients vs 0 of 870 patients; *p* < 0.001), but not with leptomeningeal disease (hazard ratio, 1.14; 95% CI, 0.73–1.77; *p* = 0.56). Prabhu et al. [68] characterized the pattern of intracranial recurrence in 147 patients who developed LMD following surgery and postoperative SRS for at least one brain metastasis. At a median time from postoperative SRS of 5.6 months, 42.9% of patients presented with classical LMD, while 57.1% presented with nodular LMD, defined as new focal extra-axial distinct nodular enhancing lesions located on the meninges or ependyma. Within the nodular LMD, the median number of nodules was two and the median distance between the surgical corridor and the closest nodule was 2 cm, with about 70% of patients having LMD nodules within 5 cm of the surgical corridor. Patients with nodular LMD had significantly longer median overall survival than those with classical LMD (8.2 vs. 3.3 months, *p* < 0.001). A new classification for intracranial progression which takes into account this peculiar pattern of intracranial nodular LMD following postoperative SRS together with classical LMD, local parenchymal recurrence, and distant intraparenchymal metastases has been suggested by these authors.

The increased shift in the pattern of intracranial recurrence after surgery and postoperative SRS to surgical bed raises the question on the optimal postoperative radiation technique for these patients. Even though adjuvant WBRT is associated with a lower risk of LMD and better local control compared to postoperative SRS, especially in case of large cavities [4, 7, 52, 69], it should be noted that randomized studies have not identified a survival benefit with WBRT for either resected or intact metastases [2, 3, 7]. Therefore, the use of postoperative SRS remains a reasonable approach to avoid neurocognitive decline associated with the use of WBRT. Future studies need to evaluate the impact of different focal radiation approaches to postoperative surgical bed in terms of dose delivery and target delineation, with the aim of reducing the high risk of “near target” localized pachymeningeal seeding, while maintaining the superiority of the SRS/HSRT approach on neurocognitive function and quality-of-life outcomes compared to WBRT. Additionally, data on outcomes of salvage treatment are needed to learn how to optimally treat patients with different patterns of intracranial progression.

### Risk of radiation necrosis

Radiation necrosis is the most significant adverse effect of radiation treatment of brain metastases. Radiation necrosis presents as a focal enhancing lesion at a variable time of 6–15 months following SRS/HSRT; however, the differential diagnosis between tumor progression and radiation necrosis remains challenging. While pathological confirmation remains the gold standard for diagnosis, non-invasive imaging techniques, including perfusion-weighted MRI and PET using amino acid tracers 11C-methionine (ref), O-(2-18F-fluoroethyl)-l-tyrosine (18F-FET) and 6-18-F-fluoro-l-dopa have emerged as highly sensitive diagnostic tools for distinguishing radiation necrosis from tumor recurrence [70, 71]. Current treatments for symptomatic radiation necrosis include corticosteroids, surgery, bevacizumab, and hyperbaric oxygen. The 12-month estimated risk of radiation necrosis following postoperative radiation of brain metastases ranges from 1.5% to 28% being similar after postoperative SRS and HSRT (Tables 1, 2); however, HSRT is typically delivered to much larger resection cavities. Although some retrospective series report radiological changes suggestive of radiation necrosis in more than 20% of patients treated with postoperative radiation, the 12-month estimated risk of symptomatic radiation necrosis is about 5–10% in the majority of studies. In a systematic review and meta-analysis on postoperative SRS following excision of brain metastases, Akanda et al. [6] observed a similar incidence of less than 10% in 28 out of 36 studies using different imaging modalities. Although there is no head-to-head comparison of postoperative HSRT versus single-fraction SRS to the surgical bed, the relatively low risk of radiation necrosis after HSRT for volumes larger than 20–25 ml suggests that hypofractionation may represent a better approach for large cavities [19, 24, 31, 32]. A risk of radiation necrosis less than 10% has been generally observed in studies of HSRT using either 24–27 Gy given in three fractions or 30–35 Gy given in 5 fractions, corresponding to an equivalent dose in 2 Gy fractions of 62–78.7 Gy using an alpha/beta of 2 Gy (EQD2_2_), and to a BED_2Gy_ of 124–157.5 Gy (Table 3). In a series of 45 consecutive patients who received fractionated partial brain radiation therapy to the surgical cavity (30–42 Gy in 3-Gy per fraction) at the Massachusetts General Hospital between April 2012 and September 2017, Byrne et al. [72] reported 12-month freedom from local failure rates of 88.2% with no events of late radiation necrosis.

Factors correlated with an increased risk of radiation necrosis include higher radiation dose, larger volumes, and combined immunotherapy [24, 29, 30, 34, 73]. Several studies have found a significant correlation between volume of brain receiving high-dose irradiation and the risk of radiation necrosis after either SRS or HSRT for intact and resected brain metastases [74–76]. For patients undergoing SRS, the volume of normal brain receiving 12 Gy (brain minus GTV; V12 Gy) > 5–10 ml is predictive of a > 10% risk of radiation necrosis [77, 78]. In the context of HSRT, volumetric constraints for brain predicting the risk of radiation necrosis include V18 Gy and V24 Gy for 3-fraction regimens and V25 Gy for 5-fraction regimens [24, 34, 78, 79]. In a recent review of single- and multifraction SRS dose/volume tolerances of the brain including 51 studies published from January 1995 through December 2016, Milano et al. [78] reported brain volumes (brain plus target volume) receiving 20 Gy in 3-fractions or V24 Gy in 5-fractions < 20 ml were associated with < 10% risk of any necrosis or edema in patients with brain metastases.

In a series of 101 patients with brain metastases treated with surgery and postoperative SRS (9 Gy × 3) to the resection cavity at University of Rome Sapienza, Sant’Andrea Hospital, the V24 Gy calculated as normal brain less GTV was the most significant factor associated with the development of radiation necrosis. The crude risk of radiation necrosis was 16% for V24 Gy ≥ 16.8 ml and 2% for V24 Gy < 16.8 ml (*p* = 0.03), with respective 12-month risk of 8% and 3% [24]. No other factors, including histology, site of tumor, PTV, and conformity index were predictive of radiation necrosis. In another series of 289 consecutive patients who received SRS or HSRT (9 Gy × 3) for at least one brain metastasis > 2.0 cm as primary treatment at Sant’Andrea Hospital, University of Rome Sapienza, the 1-year cumulative incidence rate of radionecrosis was 18% for patients undergoing SRS and 9% for those receiving HSRT (*p* = 0.01), respectively. For patients receiving HSRT, the V18 Gy was the most significant prognostic factor for radiation necrosis; the incidence was 5% for V18 Gy ≤ 30 ml and 14% for V18 Gy > 30 ml (*p* = 0.04).

In another series of 187 consecutively treated patients with 118 surgical cavities and 132 intact metastases treated with HSRT (30 Gy in 5 fractions), Faruqi et al. [34] showed that the total brain minus gross tumor volume (GTV) receiving 30 Gy (V30) was a significant risk factor for symptomatic radiation necrosis with a threshold of 10.5 ml or more (OR 7.2; *p* = 0.02). The 1-year symptomatic radiation necrosis rate was 13% for V30 < 10.5 ml and 61% for V30 ≥ 10.5 ml. In a multi-institutional retrospective review of 117 brain metastases from 83 patients treated with 5 fraction HSRT, Andruska et al. [79] found a two-year risk of symptomatic radiation necrosis of 21% for V25 > 16 ml and V30 > 10 ml and 2% for V25 ≤ 16 ml and V30 ≤ 10 ml (*p* = 0.007). In another series of 55 resected brain metastases that were treated postoperatively with HSRT (25–35 Gy in 5 fractions), Tanenbaum et al. [80] observed a 1-year incidence of radiation necrosis of 18.2%; hotspots within the PTV expansion margin > 105% and an absolute dose of 33.5 Gy were significantly associated with the development of radiation necrosis, but hotspots within the CTV did not.

### Future directions

The role of SRS in patients with resected brain metastases will continue to evolve. Postoperative SRS to the resection cavity has become the standard of care after surgery, as it provides local control rates comparable to WBRT, better than with surgery alone, and without a negative impact on survival; however, a few studies have suggested worse local control for large brain metastases after SRS compared to WBRT [7, 81]. Future research needs to evaluate the impact of different dose and fractionation on the surgical bed in terms of brain control and risk of radiation necrosis, especially for large volumes that are apparently associated with worse local control following SRS. In this regard, it will be important to compare this approach with alternative strategies, such as fractionated partial brain RT with more generous GTV-to-CTV/PTV margins or WBRT with hippocampal avoidance. A phase III trial of post-surgical single fraction SRS compared with HSRT for resected metastatic brain disease evaluating the time to surgical bed failure as primary endpoint is currently recruiting patients in the US (ClinicalTrials.gov, NCT04114981). Other critical areas of research include understanding the pattern of LMD spread and the optimal timing of adjuvant SRS since surgical cavities undergo morphological changes dependent on the time from surgery.

New strategies to enhance local control and minimize the risk of leptomeningeal disease include pre-operative SRS and the use of systemic agents, alone or in combination with radiation therapy. The rationale for pre-operative SRS is to treat tumor cells prior to potential iatrogenic dissemination at the time of surgical resection, potentially decreasing the rate of leptomeningeal disease. In addition, contouring an intact tumor for pre-operative SRS is much less challenging than for a resection cavity and if no added margin is needed, this approach may result in lower risk of radiation necrosis. In this regard, a few studies have demonstrated the safety and efficacy of preoperative SRS, reporting local control rates of 80 to 90% at 1 year with, with respective risk of symptomatic radiation necrosis and development of leptomeningeal disease of 5–10% [82–85]. Two prospective trials randomizing patients undergoing pre-operative SRS versus post-operative are currently recruiting patients (ClinicalTrials.gov, NCT03741673 and NCT03398694).

## Conclusions

Overall, just as our paradigm has shifted from WBRT to SRS for patients with a limited number of intact brain metastases, postoperative SRS is replacing WBRT for patients with resected brain metastases as the standard of care. The rationale for delivering focal radiation and not WBRT is to avoid the complications of WBRT while maintaining high local control without negatively impact on survival. Certainly, MRI at regular intervals of 2–3 months is mandatory to offer appropriate salvage therapy in the event of either local or distant brain progression. While both SRS and HSRT have been shown to improve local control in smaller surgical beds, achieving excellent local control rates still remains a challenge in larger ones. Accurate localization and delineation of the surgical cavity after resection of a brain metastasis is a crucial step in the treatment planning process for improving local control. A summary of recommended imaging modalities for target volume delineation and dose fractionation using either HSRT or SRS is reported in Table 4. Future research is needed to answer several questions regarding the optimal treatment timing, target delineation, dose/fractionation, and combination with systemic agents.

**Table 4:** Summary of imaging modalities for target volumes delineation and dose/fractionations for postoperative resection cavity of brain metastases

Imaging for target delineation	Isotropic post-contrast-enhanced 3D T1-weighted MRI sequences with 1 mm thick slices and T2-weighted images. Additional images include preoperative contrast-enhanced T1-weighted MRI sequences to identify the preoperative tumor extent and dural involvement
Gross Tumor Volume (GTV)	Surgical cavity on postoperative contrast-enhanced T1-weighted MR images (typically represented by the rim of enhancement at the edge of the resection cavity) with inclusion of any residual nodular enhancement
Clinical Tumor Volume (CTV)	The CTV is defined as the GTV plus 0–1 mm margins constrained at anatomical barriers such as the skull. GTV-to-CTV margins up to 5–10 mm are applied along the bone flap/meningeal margin, with larger margins used for tumors in contact with the dura preoperatively. Vasogenic edema and surgical corridor (for deep lesions) are not usually included
Planning Target Volume (PTV)	A margin of up to 3 mm is usually added to the CTV to generate the PTV, depending on the radiation technique. For frame-based SRS, no additional safety margin is necessary; with frameless SRS and SRT, a GTV-to-PTV safety margin of 1–3 mm is usually applied according to Institutional practice
Timing of treatment	There is a general consensus to perform postoperative SRS/HSRT to the resection cavity within 4 weeks after surgery with planning MRI acquired < 7 days before treatment to limit negative impact of cavity changes on clinical outcomes
Dose and fractionation	12–18 Gy using single-fraction SRS; 24–27 Gy in 3 fractions and 30–35 Gy in 5 fractions using HSRT, typically for larger resection cavity; less commonly 30–40 Gy in 10 fractions

SRS, stereotactic radiosurgery; HSRT, hypofractionated stereotactic radiation therapy; MRI, magnetic resonance imaging; 3D, 3-dimensional

## Data Availability

All data supporting the results of this review are published in the cited references.

## Abbreviations

BED: Biological equivalent dose
CK: CyberKnife
CNS: Central nervous system
CSF: Cerebrospinal fluid
CTV: Clinical target volume
EQD2_2_: Equivalent doses in 2 Gy fractions
GK: Gamma Knife
GTV: Gross tumor volume
HSRT: Hypofractionated stereotactic radiotherapy
LINAC: Linear accelerator
LMD: Leptomeningeal disease
KPS: Karnofsky performance status
MRI: Magnetic resonance imaging
PET: Positron-emission tomography
PTV: Planning target volume
SRS: Stereotactic radiosurgery
WBRT: Whole brain radiation therapy
